# New and Improved Nanomaterials and Approaches for Optical Bio- and Immunosensors

**DOI:** 10.3390/bios13040443

**Published:** 2023-03-31

**Authors:** Boris B. Dzantiev

**Affiliations:** A.N. Bach Institute of Biochemistry, Research Center of Biotechnology of the Russian Academy of Sciences, Leninsky Prospect 33, 119071 Moscow, Russia; dzantiev@inbi.ras.ru

The current state in the development of biosensors is largely associated with the search for new approaches to simplify measurements and lower detection limits. Nanotechnologies have provided various tools to achieve these goals; however, their comparative assessment and the reasonable choice of the most effective nanomaterials are still in demand. The use of nanoparticles as carriers and labels has good practical prospects due to the availability of simple tools for their optical registration.

The above-listed reasons have driven our interest in preparing this Special Issue. It is focused on new approaches for highly sensitive bio- and immunosensors with nanodispersed labels and their optical registration. The 11 articles of this Special Issue demonstrate the current state of developments and their most promising directions. 

Yuliya A. Podkolodnaya et al. characterize composites of carbon nanostructures and silica particles in their review [1]. This combination integrates specific features of both compounds and allows for different assay formats and detection techniques. The publication reports successful applications of the given composites for bioimaging, chromatography, immunoassays, and other important analytical tasks.

Jia Zhang et al. studied the transformation of mycotoxin sterigmatocystin upon processing contaminated rice wine [2]. They used HPLC–MS/MS to investigate changes in sterigmatocystin content and profiles of its metabolites for different rice leaven levels and fermentation times. The obtained data help enable the evaluation of health risks and finding methods for their prevention.

Galina V. Presnova et al. considered the digital detection of nucleic acids on silicon microchips based on the counting of gold nanoparticles in DNA duplexes by scanning electron microscopy [3]. This approach demonstrated high sensitivities (down to 0.04 pM for short oligonucleotides) and was effective for productive testing of genetic variability. The practical potential of the developed technique was confirmed by multiplex quantification of bacterial genes responsible for resistance against β-lactam antibiotics.

An original way to detect low quantities of pathogen-specific immunoglobulins in serum samples for efficient serodiagnosis of infection deceases is proposed in the paper by Dmitriy V. Sotnikov et al. [4]. The authors improved traditional lateral flow test strips via the two-stage incorporation of nanoparticles into immune complexes. This enables increasing the number of the detected markers per one formed immune complex (by more than two orders of magnitude), thus providing the detection of specific antibodies at low concentrations. The proposed approach was successfully applied for the serodiagnosis of COVID-19.

Oleg L. Bodulev et al. combined a chemiluminescent enzyme-linked oligonucleotide assay with mismatched catalytic hairpin assembly amplification [5]. The case study of this approach for microRNA-155 assay demonstrated a low detection limit of 400 fM. The efficiency of the assay and its perspectives for medical purposes was demonstrated by measurements of microRNA-155 content in human cancer lines.

The paper of Zhaodong Li et al. demonstrates the successful application of hapten design techniques aimed at the modulation of immunodetection specificity [6]. Due to the variety of structurally similar sulfonylurea-based anti-diabetic drugs, broad-specific immunodetection is in demand. The authors present newly produced antibodies and their use in a multiplexed enzyme-linked immunosorbent assay of sulfonylurea adulterants in functional pills.

Larisa V. Sigolaeva et al. considered new approaches for highly sensitive surface-enhanced Raman spectroscopy (SERS) [7]. Their study presents a laser-induced aggregation of silver nanoparticles and the thermoresponsive micelle-forming diblock copolymer of 1,2-butadiene and N,N-dimethylaminoethyl methacrylate. Using 4-mercaptophenylboronic acid as a reporting probe led to the laser-induced enhancement of a SERS signal. The proposed mechanism of this effect was confirmed by theoretical simulation.

Simone Cavalera et al. investigated cases of nonmonotonic dependence of the recorded signal on the analyte concentration (“hook effect”) in a lateral flow sandwich immunoassay [8]. The authors explained this effect as due to antigen saturation caused by the excess of the labeled antibody and studied the systematic variation in the immunoassay parameters that affect rapid interactions in lateral flow tests. The obtained results allowed for the development of an effective assay for lumpy skin disease virus detection.

Mariia V. Samodelova et al. developed a SERS-based sensor for detecting the SARS-CoV-2 virus using aptamers as recognizing molecules [9]. The formed sandwich complexes of the primary aptamer at the plasmonic surface, antigen-coated silver nanoparticles, and fluorophore-labeled secondary aptamer allowed an investigation of the impact of the distance between the SERS-active compound and the quencher and proposal of a protocol for high-sensitive detection.

One more SERS-based assay was developed in the study of Artem Tabarov et al. [10]. Influenza viruses have no characteristic spectral peaks and, therefore, are difficult to identify by SERS-based sensors. The authors successfully applied machine learning technologies and provided differentiation of samples containing influenza A and B viruses with high accuracy. The given development contributes to more effective medical diagnostics and assessment of epidemiological situations.

In the study by Olga E. Eremina et al., the development of molecular immobilization and resonant Raman amplification by a complex-loaded enhancers (MIRRACLE) sensor to measure intense Raman signals on a nanostructured silver-based substrate was reported [11]. The authors synthesized plier-like ligands with different functional groups as tools for shifting the maximum absorbance of catecholamines and generating a new absorbance band. The concentration range reached for the determination of dopamine was 3.2 pM–10 nM, which confirmed its prospects in the evaluation of neurotransmitter metabolism disorders.
